# The Long-Term Effects of COVID-19 Stress on Mental Health and Identity Among College Students

**DOI:** 10.3390/bs16010069

**Published:** 2026-01-04

**Authors:** Ellie Mitova, Erick Z. Negron, Lexi Bratek, Alyssa Leong, Steven L. Berman

**Affiliations:** 1 Department of Psychology, College of Sciences, University of Central Florida, Orlando, FL 32816, USA; lexi.bratek@ucf.edu (L.B.); al159762@ucf.edu (A.L.); steven.berman@ucf.edu (S.L.B.); 2 Department of Chemistry, College of Sciences, University of Central Florida, Orlando, FL 32816, USA; erick.negron@ucf.edu

**Keywords:** COVID-19, obsessive–compulsive symptoms, generalized anxiety, stress, identity distress, identity

## Abstract

The COVID-19 pandemic had widespread psychological effects, prompting research into long-term impacts on mental health and identity development. This retrospective study examined how pandemic-related stress affected obsessive–compulsive symptoms (OCS) and generalized anxiety symptoms across three timepoints, prior to the pandemic (2019–February 2020), during the height of the pandemic (March 2020–2022), and the present (within the past month), and how changes in these symptoms relates to identity. The sample consisted of undergraduate students (*N* = 476) who completed an anonymous online survey battery. Indices of psychological “damage” and “recovery” showed although OCS levels returned to baseline in the current period, anxiety levels remained elevated. COVID-related stress predicted higher OCS and anxiety symptoms across timepoints. Greater symptom damage was associated with more identity disturbance, while recovery from anxiety was related to reduced identity disturbance. Recovery from OCS was uniquely related to higher identity consolidation. These findings suggest the psychological toll of the pandemic extends beyond clinical symptoms, impacting foundations of identity. Although some psychological recovery has occurred, lingering anxiety symptoms may continue to affect developmental outcomes. Further research is needed to understand mechanisms that support long-term recovery and identity formation in the wake of large-scale stressors like the COVID-19 pandemic.

## 1. Introduction

### 1.1. Impact of the COVID-19 Pandemic on Mental Health

The Coronavirus disease 2019 (COVID-19) pandemic produced an immense impact on the world and took a toll on many people’s physical and mental health. Negative effects were evident in the public, including feelings of isolation due to quarantine, increased health-related anxiety, and financial stress. A systematic review by Xiong et al. (2020) underscored the widespread mental health challenges faced globally during this time. These results revealed notably high rates of anxiety symptoms, psychological distress, depression, and obsessive–compulsive tendencies across various populations. These stressors created a unique and chronic context of psychological strain, with implications that may extend far beyond the initial timeline of the outbreak.

As a fundamental survival mechanism, stress prompts the body to react quickly to urgent or perceived threats, a function that is essential (Montgomery & Gouvea, 2024). While most existing research on the pandemic has understandably focused on acute stress, or short-term reactions to immediate challenges (Vindegaard & Benros, 2020; Xiong et al., 2020), less attention has been given to the potential long-term psychological impacts of the prolonged and ongoing exposure to pandemic-related stressors. Furthermore, much of the current literature of the impact of the pandemic has concentrated on individuals with pre-existing clinically diagnosed conditions (Murphy et al., 2021). People with prior mental-health conditions such as obsessive–compulsive disorder (OCD), post-traumatic stress disorder, and generalized anxiety disorder (GAD) displayed an increase in symptoms during the pandemic. One study found a significant worsening in OCD symptom severity during quarantine, particularly among individuals with contamination-related fears (Vindegaard & Benros, 2020). Similarly, patients with existing psychiatric disorders reported deteriorating mental health, and COVID-19 survivors exhibited alarmingly high rates of post-traumatic stress and psychological distress (Davide et al., 2020). Even though these results signify the mental health impacts seen in individuals with pre-existing psychological diagnosis, a critical gap in the literature remains with far fewer studies examining the pandemic’s impact on the mental health of the general population.

An important segment of the general population are emerging adults, typically defined as individuals aged 18–25 who are navigating major developmental milestones such as autonomy, identity formation, and life direction decisions (Arnett, 2007). Although the term emerging adulthood was introduced by Arnett, its core developmental themes align with Erikson’s (1968) theory, which emphasizes that the transition from adolescence into adulthood involves continuous adaptability as individuals work toward a more coherent and stable sense of self. College students, who represent a large portion of emerging adults, experience developmental and life changes during this time period, struggling with an increased or decreased sense of self. As many of today’s college students were in late adolescence or emerging adulthood when the COVID-19 pandemic began, they may have experienced these disruptions during a particularly sensitive developmental period. Although the present study does not assess mental health during the pandemic itself, understanding how current students recall and interpret their pandemic-related stress remains critical. A retrospective study by Hu et al. (2022) found that college students continued to report high levels of fear, stress, and reduced well-being even two years after the outbreak, indicating that pandemic stress can have lasting psychological and academic effects on college students. Retrospective stress appraisals can reveal persistent psychological and developmental effects, especially given that emerging adulthood is marked by heightened vulnerability to stress and ongoing identity formation. Accordingly, examining this population offers insight into the potential long-term or lingering impacts of pandemic-related stressors.

### 1.2. COVID-19 and Obsessive–Compulsive Symptoms

According to the DSM-5-TR (American Psychiatric Association, 2013), OCD is a mental disorder that is diagnosed when an individual experiences obsessions, compulsions, or both. Obsessions are defined as intrusive, unwanted thoughts, urges, or images that cause significant anxiety or distress, while compulsions are repetitive behaviors or mental acts that an individual feels driven to perform in response to an obsession or according to rigid rules (American Psychiatric Association, 2013). For a clinical diagnosis, these obsessions or compulsions need to be time-consuming or cause clinically significant distress or impairment for the individual. However, a person may experience obsessive–compulsive symptoms (OCS) without meeting full diagnostic criteria for a diagnosis (Julien et al., 2007). Research has shown that individuals in the general population frequently experience intrusive thoughts that are similar in content to the obsessions observed in clinical OCD cases (Berry & Laskey, 2012; Clark et al., 2014). Common obsessions include themes such as checking, contamination, aggressive and sexual thoughts, symmetry, and scrupulosity or religiosity. While common and typically benign in the general population, under certain conditions these symptoms may intensify or become more distressing, even in individuals without a formal OCD diagnosis.

One such condition was the onset of the COVID-19 pandemic, which has been associated with a noticeable increase in OCD across clinical populations. Contamination-related obsessions became more frequent and severe during the early stages of the pandemic. AlDandan et al. (2023) found that during the pandemic, approximately 42% of participants with diagnosed OCD reported increased frequency and intensity of obsessions. Similarly, Tükel et al. (2023) found that 60% of OCD patients experienced worsening symptom severity during the pandemic. Further studies have shown that specific subtypes of OCD, particularly those related to hygiene and contamination, were especially vulnerable to environmental triggers like the pandemic. D’Urso et al. (2023) concluded that the nature of OCD is environment-dependent, with symptoms adapting in form, if not necessarily in severity. Khosravani et al. (2021) found that COVID-19 stress responses predicted increases in a wide range of OCD dimensions, including contamination fears, responsibility for harm, unacceptable thoughts, and symmetry. Collectively, these findings suggest that pandemic stress is not only a trigger but also an amplifier of OCS.

Adding a more nuanced perspective, Meșterelu et al. (2021) proposed that OCSs may serve as both protective and vulnerability factors in a pandemic. While some individuals with OCD may have benefited from their compulsions (e.g., handwashing) by reducing perceived infection risk, others experienced heightened distress. Sharma et al. (2024) found that individuals who maintained adherence to OCD treatment did not show significant increases in symptom severity compared to pre-pandemic levels, implying that treatment may serve as a protective factor. However, many individuals with OCD may not have been in treatment and therefore were more susceptible to symptom exacerbation.

### 1.3. COVID-19 and Anxiety

Generalized anxiety disorder (GAD) is classified by large amounts of worry or anxiety that takes place over a period of 6 months of more (American Psychiatric Association, 2013). GAD symptoms have shown a notable increase during the pandemic. AlDandan et al. (2023) reported that nearly half of individuals with OCD also reported increased anxiety symptoms during the pandemic. Tükel et al. (2023) further demonstrated that fear and obsession with COVID-19 were closely linked to heightened anxiety levels, regardless of whether participants had an OCD diagnosis. These findings align with broader trends observed during the pandemic, suggesting that the psychological toll extended beyond specific clinical populations. Zhu et al. (2023) further found that the overall prevalence of both anxiety and depression increased during the pandemic. This points to a general trend of worsening mental health symptoms even in people who had no diagnosis before the COVID-19 outbreak. Anxiety post COVID-19 has become so prevalent that conditions such as “post COVID-19 syndrome” have been designated to help describe the intricate relationship between anxiety and COVID-19 (Burkauskas et al., 2024).

Olgun Yıldızeli et al. (2023) also found that not only was anxiety prevalence higher in those post COVID-19 but that the anxiety symptoms were more severe in those who had the virus than those who did not. This anxiety increase, when combined with the overall trend of increased anxiety in college students, can produce detrimental effects to students’ academic and social lives (Tan et al., 2023). This compounded effect may intensify the challenges students face in managing academic responsibilities and maintaining social connections. Research has also found that ongoing increases in anxiety have been linked to poor academic performances from students (Mazzone et al., 2007). Lisnyj et al. (2020) showed that this decrease in academic performance was most likely caused by the effect of anxiety on memory and cognitive function. The ongoing rise in anxiety and its negative impact on academic performance has raised significant concerns about the long-term well-being and success of students.

While most research has highlighted symptom escalation during the pandemic, fewer studies have tracked recovery or remission in anxiety post-pandemic, particularly in young adult populations. Understanding these patterns is critical for long-term mental health planning at colleges and universities. This study aimed to identify these COVID-19-related anxiety effects to determine long term side effects of the pandemic.

### 1.4. Stress and the Impact on Identity

Stressful life events, practically those that disrupt a person’s sense of stability or direction, can significantly influence the process of identity development. These developmental implications are particularly salient for emerging adults, for whom identity exploration and consolidation are central developmental tasks (Arnett, 2007). In Erikson’s framework, this period corresponds to the stage of identity versus role confusion, during which individuals work to form a more coherent sense of self (Erikson, 1968). As college students are situated squarely within this developmental period, stressful life-events have a heightened potential to disrupt their identity development due to the academic, social, and personal transitions during a time of heightened identity sensitivity.

Such disruptions can interfere with the formation of a coherent self-concept, often resulting in what is referred to as identity distress or disruption. Berman et al. (2004) defined identity distress as psychological discomfort individuals may experience when struggling to define or comprehend important aspects of their sense of self. Identity disruption is a similar concept and is a loss of identity integration following a serious life event (Mitchell et al., 2020). Scott et al. (2014) showed that identity distress can be caused by serious events, or even previous psychological diagnoses such as PTSD. Identity disturbance has also been linked to COVID-19. Many studies have shown that the pandemic had a positive relationship with identity distress and disturbance (Obrenovic et al., 2024). Obrenovic et al. (2024) further found that increases in anxiety were closely linked to heightened identity disturbance. Similarly, Meca et al. (2023) demonstrated that the effects of COVID-19 on identity distress also negatively impacted academic adjustment, highlighting the pandemic’s long-term influence on the student population. Additionally, Wagaman et al. (2022) reported that stress and COVID-19-related anxiety had significantly exacerbated identity distress, with these symptoms being particularly pronounced among first-generation immigrant students. Taken together, these findings suggest that the prolonged stress of the COVID-19 pandemic may have substantial and lasting implications for identity development.

Another aspect of identity potentially impacted by COVID-19 is consolidated identity. Consolidated identity has been found to be negatively correlated with disturbed identity and is when an individual is more stable and secure in their identity (Kaufman et al., 2019). Mitchell et al. (2024) found that during COVID-19 identity disturbance and distress was not the only aspect of identity that was changing. They also found that during COVID-19 some adults experienced positive changes to identity such as revising their assessment of strengths and weaknesses. Although these are changes in identity, these impacts would be more related to identity consolidation and could be considered potential benefits of the stressors experienced during COVID-19.

Despite the potential benefits of stressors, most studies have consistently shown that COVID-19 was a significant stressor that disrupted many people’s lives, with identity playing a crucial role in how individuals coped with this challenge (Harris et al., 2024). With all the impacts COVID-19 had on the general population, it is important for research to continue to try and find the long-term effects of the pandemic, with respect to identity development or disruption.

### 1.5. Rationale

In line with previous dimensional perspectives, the current study focused on OCS severity rather than formal diagnoses as well as generalized anxiety symptoms rather than a formal diagnosis. By examining symptoms along a continuum, this approach captures subclinical experiences that may still contribute to psychological distress, particularly during times of widespread stress, such as a global pandemic. This is especially important because many individuals with significant symptoms do not receive formal diagnoses or treatment. Furthermore, the present study employs a retrospective longitudinal design, assessing both OCS severity as well as generalized anxiety symptoms at three key timepoints: prior to the pandemic (2019–February 2020), during the height of the pandemic (March 2020–2022), and the present (within the past month). This allows for a more nuanced understanding of how symptoms may have increased, persisted, or recovered over time, offering insight into the long-term mental health consequences of COVID-19-related stress. Even though the study assesses psychological symptoms over these three time periods, all data was collected at a single point in time, hence the retrospective design of the study. In addition to tracking symptom trajectories over time, it is also critical to consider how elevated OCD and anxiety symptoms may intersect with identity development, particularly in college students. As a result, high symptom severity, even in the absence of a formal diagnosis, may interfere with students’ ability to engage in identity development, potentially leading to long-term difficulties in self-esteem, purpose, and psychological well-being.

By evaluating the psychological outcomes associated with the COVID-19 pandemic, this study sought to clarify the long-term impact of pandemic-related stress on mental health, particularly focusing on obsessive–compulsive symptom severity, anxiety, and identity development. These findings aim to contribute to a better understanding of how large-scale public health crises influence psychological resilience and vulnerability. Ultimately, this research may inform targeted interventions and mental health strategies for future global disruptions.

Specifically, the study hypothesized that

1. OCS severity & Anxiety symptoms would increase from prior to the pandemic (2019–February 2020) to during the height of the pandemic (March 2020–2022).
2. OCS severity & Anxiety symptoms would show a decrease from during the height of the pandemic (March 2020–2022) to present time (within the past month) but would not have returned to as low as prior to the pandemic symptoms (2019–February 2020).
3. Higher levels of COVID-related stress would be significantly associated with greater increases in both OCS and anxiety symptoms during the height of the pandemic, and with less recovery in both symptom domains over time.
4. Changes in OCS severity and Anxiety symptoms would be related to identity factors such that both the damage done by COVID (changes from prior to the pandemic to during the height of the pandemic) as well as recovery (changes from during the height of the pandemic to present) would predict identity development factors (consolidated identity, identity distress, disturbed identity, and lack of identity); however, recovery would have the greatest effects on identity.

## 2. Materials and Methods

### 2.1. Participants

Participants (*N* = 476) were undergraduate students that were recruited from a major metropolitan university in the southeastern region of the United States. The university serves a predominately commuter student population with over 60,000 full-time students. To participate in this study, participants must have been at least 18 years old, currently enrolled as full-time undergraduate students, registered with the university’s SONA system, and enrolled in psychology courses. No additional exclusion criteria were applied. Participants were enrolled in general psychology courses who accessed the study through the university’s online SONA research participation system. They voluntarily selected this study from a list of available options and completed it in exchange for course credit. Validity checks were embedded throughout the survey to ensure attentive and consistent responding. Of the total sample, 61.7% identified as female, 36.8% identified as male, 1.3% identified as non-binary, and 0.2% identified as “other”. Although most participants were traditional-aged college students, the university enrolls a substantial number of nontraditional and returning adult learners, explaining the broader upper age range. Participants’ age ranged from 18 to 68 years old (*M* = 20.54, *SD* = 5.55). Most of the participants were freshmen (40.9%), 23.3% were sophomores, 23.1% were juniors, 12.2% were seniors, and 0.4% were non-degree seeking. The sample was ethnically diverse with 47.7% identifying as White or Caucasian, 25.5% as Hispanic or Latino/a, 9.4% as Black or African American, 7.9% as Asian or Pacific Islander, and 9.4% as mixed origin or other.

### 2.2. Measures

A demographic questionnaire included questions related to age, gender identity, ethnicity, and college grade.

The Yale–Brown Obsessive Compulsive Scale was used as a self-report tool to assess the severity of obsessive–compulsive symptoms, not as a clinical OCD diagnostic tool (Goodman, 1989). Participants were asked to select responses to 10 multiple-choice items designed to measure the severity of their obsessive thoughts (items 1–5) and compulsive symptoms (6–10). To assess obsessive–compulsive symptoms over time, this scale was administered three times throughout the survey, asking (I) pre-COVID-19 pandemic (2019–February 2020), (II) during the COVID-19 pandemic (March 2020–2022), and (III) within the last month that the study was administered. For each time period, participants were asked to reflect on their obsessive–compulsive symptoms to the best of their knowledge. In the present study, Cronbach’s alphas were 0.86 for pre- COVID-19 pandemic, 0.91 for during the COVID-19 pandemic, and 0.90 at the time of taking the survey for the total obsessive–compulsive symptoms throughout each time period. This self-report version has demonstrated strong convergent validity with other measures of OCD symptoms, including the clinician-administered Y-BOCS-II, and fair discriminant validity from unrelated constructs such as depression and general impairment, supporting its specificity in assessing obsessive–compulsive symptom severity (Castro-Rodrigues et al., 2018; Rowa et al., 2025). Sample items included “How much distress did your obsessive thoughts cause you?” and “How much of an effort did you make to resist the compulsions?”.

The Generalized Anxiety Disorder-7 Scale was used to assess the severity of generalized anxiety symptoms (Spitzer et al., 2006). Participants were asked to rate on a 4-point scale ranging from 1 (not at all) to 4 (nearly every day) 7 statements on whether they were bothered by common anxiety symptoms. To assess generalized anxiety symptoms over time, this scale was administered three times throughout the survey, asking (I) before the COVID pandemic (2019–February 2020), (II) during the COVID pandemic (March 2020–2022), and (III) within the last month that the study was administered. In the present study, Cronbach’s alphas were.91 for before the COVID pandemic, 0.92 for during the COVID pandemic, and 0.93 for at the time of the survey for the total anxiety symptoms throughout each time period. This measure has demonstrated strong convergent validity with other measures of anxiety, such as the Beck Anxiety Inventory (*r* = 0.72), and fair discriminant validity from the PHQ-8 depression scale, supporting its specificity in assessing anxiety symptom severity (Löwe et al., 2008; Spitzer et al., 2006). Sample items included “Trouble relaxing” and “Became easily annoyed or irritable”.

The COVID-19 Stress Scale was used to assess stress and anxiety checking symptoms related to the COVID-19 pandemic (Taylor et al., 2020). Participants were asked to rate on a 5-point scale ranging from 1 (not at all) to 5 (extremely) 24 statements on whether they experienced various kinds or worries during the COVID pandemic (March 2020–2022). They were also asked to rate on a 5-point scale ranging from 1 (never) to 5 (almost always) 12 statements on whether they experienced any anxiety-checking behaviors during the COVID pandemic (March 2020–2022). In the present study, Cronbach’s alpha was 0.97 for the total pandemic-related stress and anxiety-checking behaviors during the pandemic. This measure has demonstrated strong convergent validity with pre-COVID trait measures of healthy anxiety and obsessive–compulsive contamination, and fair discriminant validity when compared to measures assessing current anxiety and depression, supporting specificity in assessing stress and anxiety checking symptoms related to the COVID-19 pandemic (Liu et al., 2023; Taylor et al., 2020). Sample items included “If I met a person from a foreign country, I was worried that they might have the virus” and “I had trouble sleeping because I was worried about the virus”. For the sake of this study, the pandemic was referred to as “the virus”.

The Identity Distress Scale was used to assess the degree of distress participants felt concerning unresolved identity concerns (Berman et al., 2004). Participants were asked to rate how much distress they experienced in relation to 7 identity domains, including long-term goals, career choices, friendships, sexual orientation, religion, values and beliefs, and group loyalties. Participants rated the level of distress on a 5-point scale ranging from 1 (none at all) to 5 (very severely). Cronbach’s alpha was 0.81 for the average degree of identity distress currently. This measure has demonstrated strong convergent validity with related constructs of identity consolidation and distress, and good discriminant validity when compared to unrelated constructs, such as anxiety and depression, supporting its specificity in assessing identity-related difficulties (Berman et al., 2004, 2009; Hernandez et al., 2006). Sample items included “Career choice? (e.g., deciding on a trade or profession, etc.)” and “Values or beliefs? (e.g., feeling confused about what is right or wrong, etc.)”.

The Self-concept and Identity Measure was used to assess lack of identity functioning and identity disturbances (Kaufman et al., 2015. The scale has three subscales, consolidated identity, lack of identity, and disturbed identity. Participants were asked to rate on a 7-point scale ranging from 1 (strongly disagree) to 7 (strongly agree) 27 statements on whether they had struggled with certain self-concept or difficulties with identity. In the present study, Cronbach’s alpha was 0.80 for consolidated identity, 0.91 for lack of identity, and 0.90 for disturbed identity. This measure has shown robust convergent validity with constructs related to self-concept and identity development, and adequate discriminant validity from general anxiety and depression, indicating that it specifically captures identity-related functioning (Bogaerts et al., 2023; Kaufman et al., 2019). Sample items included “I know what I believe or value” for consolidated, “I am broken” for lack, and “I imitate other people instead of being myself” for disturbed.

### 2.3. Procedure

This study was reviewed and approved by the authors’ Institutional Review Board. Upon receiving approval, students who selected the study through SONA were provided with a link directing them to an anonymous online survey hosted on Qualtrics. Before beginning the survey, participants completed an informed consent form outlining the purpose of the study, the voluntary nature of participation, their right to withdraw at any time without penalty, and assurance of confidentiality. Only participants who provided electronic consent were allowed to proceed to the survey.

After providing consent, participants completed a series of self-report questionnaires measuring symptoms of anxiety, obsessive–compulsive behavior, COVID-related stress, and identity disturbance. The study used a retrospective design, asking participants to recall and report their symptoms and experiences across different time periods: before, during, and after the heights of the COVID-19 pandemic. This approach captures perceived changes over time through subjective interpretations. Participants were instructed to respond to all items as accurately and honestly as possible based on their recent experiences. The entire survey took approximately 20 to 30 min to complete. As an incentive, students received course credit for their undergraduate psychology course for their participation in the study. Those who chose not to participate in a research study were offered an alternative assignment of equal credit value.

Data analysis was conducted using IBM SPSS Statistics (version 29.0.2.0). The software was employed to perform descriptive and inferential statistical analyses, including calculations of means and standard deviations, as well as hypothesis testing. Pearson correlations, *t*-tests and regression analyses were used when appropriate. Data was initially screened for missing values and outliers to ensure the assumptions of each statistical test were met. SPSS facilitated the systematic organization and management of the dataset, allowing for efficient computation of statistical metrics for reporting.

## 3. Results

### 3.1. Preliminary Analysis and Descriptive Statistics

A series of statistical analyses were conducted to examine whether significant differences in the study variables existed across demographic groups. Pearson correlation matrix was run to determine if there were any significant associations with age and the studies variables. Age was significantly associated with several study variables. Specifically, age was inversely associated with lower identity distress (*r* = −0.12, *p* < 0.01), disturbed identity (*r* = −0.23, *p* < 0.001), lack of identity (*r* = −0.12, *p* < 0.01), consolidated identity (r = −0.12, *p* < 0.05), and anxiety-related damage (*r* = −0.13, *p* < 0.01).

An independent samples *t*-test was used to determine if there were any significant gender differences in the study variables. Females scored significantly higher than males in generalized anxiety symptoms during the height of the pandemic (*t*_(454)_ = −0.399, *p* < 0.001), generalized anxiety symptoms in present time (*t*_(454)_ = −3.30, *p* = 0.001), average COVID-19 stress (*t*_(454)_ = −3.10, *p* = 0.002), and in overall anxiety damage (*t*_(454)_ = −3.50, *p* < 0.001), whereas males scored significantly higher in disturbed identity (*t*_(454)_ = 2.77, *p* = 0.006). There were no other significant differences between genders on any other study variables.

A One-Way Analysis of Variance (ANOVA) was used to determine if there were any significant ethnic differences in the study variables. If there were any initial differences, a Scheffe Post Hoc comparison was conducted. Black or African Americans scored significantly higher than White or Caucasians in average COVID-19 stress (*F*_(4, 458)_ = 4.05, *p* = 0.003). Furthermore, a significant effect of ethnicity was found on disturbed identity (*F*_(4, 458)_ = 2.80, *p* = 0.026) and lack of identity (*F*_(4, 458)_ = 2.93, *p* = 0.021); however, post hoc comparisons did not reveal any statistically significant differences between specific ethnic groups (*p*-values > 0.05), suggesting that the overall group difference was not driven by strong pairwise contrasts. There were no other significant differences between ethnicity and the remaining study variables.

A One-Way ANOVA was used to determine if there were any significant differences in grades (“current year in school”) in the study variables. If there were any initial differences, a Scheffe Post Hoc comparison was conducted. Seniors scored significantly higher than both freshmen and sophomores in OCSs prior to the pandemic (*F*_(3,457)_ = 4.55, *p* = 0.004). Seniors also scored significantly higher than freshmen sophomores and juniors in anxiety symptoms prior to the pandemic (*F*_(3,457)_ = 8.76, *p* < 0.001). And finally, seniors scored significantly higher than sophomores in average disturbed identity (*F*_(3,457)_ = 4.04, *p* = 0.007). There were no other significant differences between grades and the remaining study variables.

### 3.2. Main Analysis

#### 3.2.1. Hypothesis 1

To determine whether OCS severity increased from prior to the pandemic (2019–February 2020) to during the height of the pandemic (March 2020–2022), a paired samples *t*-test was run between both variables. Results indicated a significant increase in OCS severity following the onset of the COVID-19 pandemic, *t*_(462)_ = −8.41, *p* < 0.001, *d* = 5.61. Specifically, participants reported lower OCS scores prior to the pandemic (*M* = 19.37, *SD* = 6.60) compared to during the height of the pandemic (*M* = 21.56, *SD* = 8.04), suggesting that the pandemic period was associated with a notable rise in obsessive–compulsive symptoms. Similarly, a paired-samples *t*-test was run between anxiety symptoms prior to the pandemic (2019–February 2020) and anxiety symptoms during the height of the pandemic (March 2020–2022), to determine whether generalized anxiety symptoms increased between both time periods. Results indicated a significant increase in generalized anxiety symptoms following the onset of the COVID-19 pandemic, *t*_(460)_ = −12.51, *p* < 0.001, d = 4.87. Specifically, participants reported lower generalized anxiety symptom scores prior to the pandemic (*M* = 12.31, *SD* = 5.07) compared to during the height of the pandemic (*M* = 15.14, *SD* = 5.70), suggesting that the pandemic period was associated with a prominent rise in anxiety symptoms. See Table 1.

#### 3.2.2. Hypothesis 2

To determine whether both OCS severity and anxiety showed a decrease from during the height of the pandemic (March 2020–2022) to present time (within the past month), while determining whether the total severity had returned to as low as prior to the pandemic levels (2019–February 2020), a repeated-measures general linear model (GLM) was conducted for each variable, separately. Time1 (prior to the pandemic) Time 2 (during the height of the pandemic) and Time 3 (present) were entered as a within-subjects factor with three levels, and outcome variables included OCS severity and anxiety.

**Figure 1:**
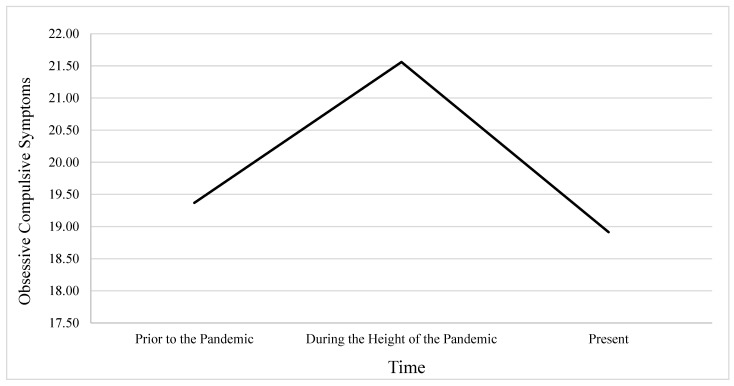
Changes in OCS Severity Over Time in Relation to the COVID-19 Pandemic.

The first GLM was conducted using OCS severity as the dependent variables. Mauchly’s Test of Sphericity indicated that the assumption of sphericity had been violated, *χ*^2^_(2)_ = 25.52, *p* < 0.001; therefore, Greenhouse–Geisser corrections were applied to the degrees of freedom. Results revealed a significant main effect of time on OCSs, *F*_(1.90, 876.78)_ = 48.98, *p* < 0.001, indicating that OCS severity significantly varied across the three time points. Estimated marginal means showed that OCSs increased from the period prior to the pandemic (*M* = 19.37, *SE* = 0.31) to the period during the height of the pandemic (*M* = 21.56, *SE* = 0.37) and then decreased at the present time (*M* = 18.91, *SE* = 0.35). Pairwise comparisons using Sidak correction indicated that the increase from prior to the pandemic to during the height of the pandemic was significant (*p* < 0.001), as was the decrease from during the height of the pandemic to present (*p* < 0.001). However, the difference between OCS scores from prior to the pandemic to present was not statistically significant (*p* = 0.276), suggesting that current symptom levels had largely returned to baseline. See Figure 1.

**Figure 2:**
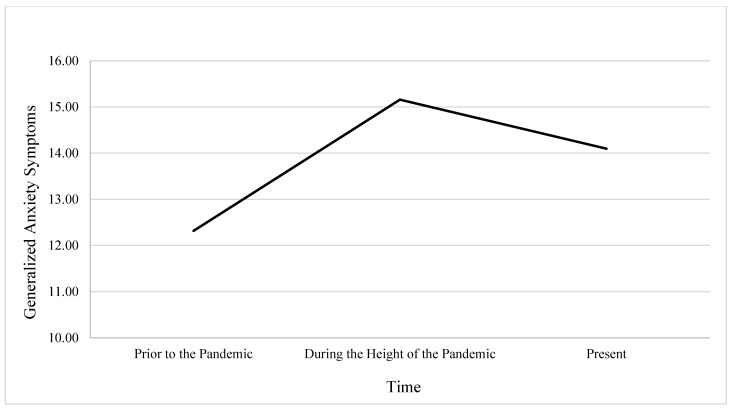
Changes in Anxiety Severity Over Time in Relation to the COVID-19 Pandemic.

The second GLM was conducted using generalized anxiety symptoms as the dependent variables. Mauchly’s Test of Sphericity approached significance, *χ*^2^_(2)_ = 6.01, *p* = 0.05; therefore, the Greenhouse-Geisser correction was applied to adjust the degrees of freedom. Results revealed a significant main effect of time on generalized anxiety symptoms, *F*_(1.97, 912.19)_ = 71.37, *p* < 0.001, indicating that generalized anxiety significantly varied across the three time points. Estimated marginal means showed that anxiety symptoms increased from the period prior to the pandemic (*M* = 12.32, *SE* = 0.24) to the period during the height of the pandemic (*M* = 15.16, *SE* = 0.27) and then decreased at the present time (*M* = 14.10, *SE* = 0.26). Although symptoms decreased slightly in the present, this level remained significantly higher than the levels prior to the pandemic (*p* < 0.001) and was also significantly lower than levels during the pandemic period (*p* < 0.001). These results suggest that while anxiety symptoms had declined since the height of the pandemic, they had not returned to baseline levels seen prior to the pandemic. See Figure 2.

#### 3.2.3. Hypothesis 3

To better understand the longitudinal change in OCS severity and generalized anxiety symptoms over time, four variables were created. Specifically, OCS Damage and Anxiety Damage were computed by subtracting symptom scores prior to the pandemic from symptom scores during the height of the pandemic, providing an index of symptom increase or “overall damage” due to the pandemic. Conversely, OCS Recovery and Anxiety Recovery were calculated by subtracting current symptom scores from symptom scores during the height of the pandemic, reflecting the degree of symptom reduction or “overall recovery” since the height of the pandemic. To test whether COVID-19-related stress predicted changes in OCS severity and anxiety symptoms over time, four separate linear regression analyses were conducted using the CSS mean score as the predictor.

In the first model, COVID-related stress significantly predicted increases in OCS severity from prior to the pandemic to during the height of the pandemic (OCS Damage), *β* = 0.04, *t*_(461)_ = 5.00, *p* < 0.001, accounting for approximately 5% of the variance, *R*^2^ = 0.05. Likewise, in the second model, COVID-related stress significantly predicted increases in generalized anxiety symptoms from prior to the pandemic to during the height of the pandemic (Anxiety Damage), *β* = 0.05, *t*_(461)_ = 7.60, *p* < 0.001, accounting for approximately 11% of the variance, *R*^2^ = 0.11.

In contrast, in the third model, COVID-related stress negatively predicted OCS recovery, with higher stress scores associated with smaller reductions in OCS severity over time, *β* = −0.04, *t*_(461)_ = −4.03, *p* < 0.001, *R*^2^ = 0.03. Similarly in the fourth model, COVID-related stress negatively predicted anxiety recovery, *β* = −0.03, *t*_(461)_ = −3.14, *p* = 0.002, *R*^2^ = 0.02. See Table 2.

#### 3.2.4. Hypothesis 4

To test the hypothesis that changes in OCS and anxiety symptoms would predict identity development outcomes, a series of hierarchical multiple regression analyses were conducted. In each model, OCS severity and generalized anxiety symptoms “damage” (i.e., the change from prior to the pandemic to during the height of the pandemic) were entered in Step 1, followed by OCS severity and generalized anxiety symptoms “recovery” (i.e., the change from during the height of the pandemic to the present) in Step 2.

For identity distress, the initial model including only damage variables was not significant, *F*_(2, 460)_ = 1.46, *p* = 0.23, *R*^2^ = 0.01. However, the addition of recovery variables significantly improved the model, *F*_(2, 458)_ = 22.70, *p* < 0.001, *R*^2^ = 0.10. Anxiety recovery emerged as a significant negative predictor of identity distress, indicating that greater recovery from anxiety was associated with reduced identity distress. In this model, OCS recovery was not a significant predictor.

For consolidated identity, the initial model with OCS and anxiety damage was not significant. When OCS recovery and anxiety recovery variables were added in Step 2, the overall model reached significance, *F*_(2, 458)_ = 6.36, *p* = 0.002, and accounted for 3% of the variance (*R*^2^ = 0.03). Notably, OCS recovery was a significant positive predictor of consolidated identity, while anxiety recovery was not a significant predictor. These results suggest that individuals who showed greater recovery in OCS were more likely to report a more consolidated identity.

For disturbed identity, the damage-only model was significant, *F*_(2, 460)_ = 3.47, *p* = 0.03, *R*^2^ = 0.02. Including recovery variables in Step 2 led to a significant improvement in model fit, *F*_(2, 458)_ = 17.60, *p* < 0.001, *R*^2^ = 0.09. Both OCS damage and anxiety damage were significant positive predictors, suggesting that symptom increases during the pandemic were related to greater identity disturbance. Additionally, anxiety recovery was a significant negative predictor, indicating that improvements in anxiety symptoms were associated with lower identity disturbance. OCS recovery was not significant in this model.

For lack of identity, the initial model with damage variables was significant, *F*_(2, 459)_ = 3.89, *p* = 0.02, *R*^2^ = 0.02. The full model, including recovery variables, showed a significant improvement, *F*_(2, 457)_ = 19.90, *p* < 0.001, *R*^2^ = 0.10. Both OCS damage and anxiety damage were significant predictors of lack of identity. Anxiety recovery was a significant negative predictor, suggesting that recovery in anxiety symptoms contributed to reduced feelings of lack of identity. OCS recovery was not a significant predictor. See Table 3.

## 4. Discussion

The aim of this paper was to establish evidence for the long-term psychological impacts of the COVID-19 pandemic, with a specific focus on obsessive–compulsive symptoms (OCS) and generalized anxiety symptoms, and how these changes relate to identity development in college students.

Consistent with our expectations, results showed that both OCS and anxiety significantly increased during the height of the pandemic as compared to levels prior to the pandemic. This is in line with previous research highlighting how uncertainty, isolation, and health-related fears during the COVID-19 pandemic likely contributed to elevated psychological symptoms, even among individuals without formal diagnoses (Tükel et al., 2023; Vindegaard & Benros, 2020; Zhu et al., 2023). Although both types of symptoms decreased after the height of the pandemic, the pattern of recovery was marginally different. Obsessive–compulsive symptoms returned to levels that were not significantly different from scores prior to the pandemic. In contrast, anxiety symptoms remained significantly elevated, even in the most recent assessment. This suggests that while some symptoms may improve as circumstances change, others, primality symptoms associated with anxiety, may take longer to fully resolve. This uneven recovery pattern aligns with research showing that anxiety can be more persistent, especially when individuals remain in unpredictable or challenging environments (Burkauskas et al., 2024; Tan et al., 2023). From a theoretical perspective, these findings can be explained through the link between anxiety and a person’s cognitive appraisal of future threats, and if someone continues to feel uncertain or overwhelmed, those symptoms may remain high even after the initial stressor has passed (Montgomery & Gouvea, 2024).

To examine the impact of the COVID-19 pandemic on mental health over time, we quantified changes in symptom levels from prior to the pandemic to during the height of the pandemic (damage) and from during the height of the pandemic to present (recovery). Surprisingly, greater pandemic-related stress was associated with larger increases in OCS and anxiety symptoms during the heights of the pandemic, as well as smaller reductions in these symptoms seen in the years that followed. In other words, people who felt more stressed by the pandemic were more likely to experience a bigger spike in OCS and anxiety symptoms during that time, and those symptoms did not improve as much afterward as compared to those who felt less stressed. These results indicate a sustained association between earlier stress exposure and later symptom levels, although the direction of this relationship cannot be determined due to the retrospective and correlational design.

Associations were also observed between psychological symptoms and current identity outcomes. Greater recovery from anxiety symptoms was consistently associated with more positive identity outcomes, including lower identity distress, disturbance, and confusion, highlighting the potential role of emotional stabilization in supporting identity formation. In contrast, OCS-related recovery showed a more limited relationship with identity outcomes, emerging only in the model predicting a more consolidated identity, while having no significant effects in other domains. Additionally, increases in both OCS and anxiety symptoms during the height of the pandemic were linked to more disturbed and confused identities in the present, suggesting that early psychological disruptions may have lasting implications for identity development in young adulthood. Together, these findings underscore the importance of considering both the psychological damage and recovery trajectories when examining the developmental impact of large-scale stressors such as the COVID-19 pandemic.

While this study provides valuable insights into the psychological impact of the COVID-19 pandemic, several limitations must be considered when interpreting the findings. Identifying the limitations of this study not only helps contextualize its findings but also guides future investigations in this area.

Primarily, the study utilized self-reported data, which may be subject to biases such as inaccurate response or recall bias. Participants may have under- or overestimated their levels of psychological symptoms, particularly when reflecting on past experiences during the COVID-19 pandemic. On the same note, the Yale–Brown Obsessive Compulsive Scale was originally developed as a clinician-administered interview rather than a self-report questionnaire. Although the self-report version is increasingly used in research and clinical contexts, this adaptation may introduce potential validity concerns through differences in symptom interpretation or reporting bias. Another limitation would be the use of a retrospective design that was employed to capture changes in perceived stress over time, rather than assessing participants longitudinally in real-time throughout each period. This retrospective reporting introduces the possibility of memory distortion, which may affect the accuracy of the reported data.

Additionally, identity was assessed only at the present time, as it is a developmental construct that evolves over time and may not be accurately captured retrospectively. This limits conclusions about changes in identity over the pandemic period and should be addressed in future studies. Furthermore, while the study quantified dynamic “damage” and “recovery” scores to capture changes in psychological symptoms over time, these scores may be influenced by measurement artifacts, regression to the mean, or pre-existing individual differences. As such, caution is warranted when interpreting these changes as true psychological change attributable solely to the COVID-19 pandemic.

Similarly, although the sample was diverse, participants for this study were drawn exclusively from introductory courses and recruited through a course-credit system, resulting in a convenience sample. Other factors, such as potential socioeconomic or academic differences may also influence both stress exposure and identity formation. As such, the sample may not fully represent the broader undergraduate population and may overrepresent college students. Therefore, caution should be used when generalizing the findings beyond similar undergraduate samples and educational contexts. Future research would benefit from utilizing longitudinal designs with diverse, representative samples and real-time data collection methods to more accurately capture psychological change and minimize recall bias.

## Figures and Tables

**Table 1:** Paired Samples *t*-Test Comparing Obsessive–Compulsive and Anxiety Symptoms from Prior to the Pandemic to During the Height of the Pandemic.

	Prior to the Pandemic (2019–February 2020)		During the Height of the Pandemic (March 2020–2022)					
	*M*	*SD*	*M*	*SD*	*df*	*t*	*p*	Cohen’s *d*
OCS Severity	19.37	6.60	21.56	8.04	462	−8.41	**<0.001**	5.61
Anxiety Symptoms	12.31	5.07	15.14	5.70	460	−12.51	**<0.001**	4.87

Note: Significant *p* values are in bold.

**Table 2:** Linear Regression Analyses Examining COVID-19 Stress as a Predictor of Obsessive–Compulsive Symptom Severity and Anxiety Symptom Change (Damage and Recovery).

Variables	COVID-19-Related Stress				
	*β*	*t*	*df*	*p*	*R* ^2^
OCS Damage	0.04	5.00	461	**<0.001**	0.05
Anxiety Damage	0.05	7.60	461	**<0.001**	0.11
OCS Recovery	−0.04	−4.03	461	**<0.001**	0.03
Anxiety Recovery	−0.03	−3.14	461	**0.002**	0.02

Note: Significant *p* values are in bold.

**Table 3:** Hierarchical Regression Results Predicting Identity Outcomes from OCS and Anxiety Symptom Changes.

Variables	Identity Distress			Consolidated Identity			Disturbed Identity			Lack of Identity		
	*β*	*t*	*p*	*β*	*t*	*P*	*β*	*t*	*p*	*β*	*t*	*p*
OCS Damage	0.05	0.87	0.390	−0.12	−1.95	0.052	0.15	2.50	**0.013**	0.19	3.19	**0.002**
Anxiety Damage	0.18	3.30	**0.001**	−0.03	−0.47	0.640	0.15	2.72	**0.007**	0.14	2.49	**0.013**
OCS Recovery	0.08	1.32	0.186	0.17	2.53	**0.012**	−0.04	−0.67	0.505	−0.10	−1.56	0.121
Anxiety Recovery	−0.37	−6.27	**<0.001**	0.05	0.75	0.454	−0.28	−4.60	**<0.001**	−0.26	−4.30	**<0.001**

Note: Significant *p* values are in bold.

## Data Availability

The data sets presented in this article are not readily available because participants were informed that data would not be shared beyond the research team as noted in the consent form.

## Abbreviations

The following abbreviations are used in this manuscript:

COVID-19	Coronavirus Disease 2019
GAD	Generalized Anxiety Disorder
OCD	Obsessive–Compulsive Disorder
OCS	Obsessive–Compulsive Symptoms
